# Bacterial genospecies that are not ecologically coherent: population genomics of *Rhizobium leguminosarum*

**DOI:** 10.1098/rsob.140133

**Published:** 2015-01-14

**Authors:** Nitin Kumar, Ganesh Lad, Elisa Giuntini, Maria E. Kaye, Piyachat Udomwong, N. Jannah Shamsani, J. Peter W. Young, Xavier Bailly

**Affiliations:** Department of Biology, University of York, York YO10 5DD, UK

**Keywords:** bacterial species, core genome, accessory genome, ecotype, phenotype

## Abstract

Biological species may remain distinct because of genetic isolation or ecological adaptation, but these two aspects do not always coincide. To establish the nature of the species boundary within a local bacterial population, we characterized a sympatric population of the bacterium *Rhizobium leguminosarum* by genomic sequencing of 72 isolates. Although all strains have 16S rRNA typical of *R. leguminosarum*, they fall into five genospecies by the criterion of average nucleotide identity (ANI). Many genes, on plasmids as well as the chromosome, support this division: recombination of core genes has been largely within genospecies. Nevertheless, variation in ecological properties, including symbiotic host range and carbon-source utilization, cuts across these genospecies, so that none of these phenotypes is diagnostic of genospecies. This phenotypic variation is conferred by mobile genes. The genospecies meet the Mayr criteria for biological species in respect of their core genes, but do not correspond to coherent ecological groups, so periodic selection may not be effective in purging variation within them. The population structure is incompatible with traditional ‘polyphasic taxonomy′ that requires bacterial species to have both phylogenetic coherence and distinctive phenotypes. More generally, genomics has revealed that many bacterial species share adaptive modules by horizontal gene transfer, and we envisage a more consistent taxonomic framework that explicitly recognizes this. Significant phenotypes should be recognized as ‘biovars' within species that are defined by core gene phylogeny.

## 2. Introduction

The species is a central concept in biology, fundamental to our description and understanding of biological diversity from the perspectives of both ecology and genetics. While dozens of definitions have been proposed, most biologists think of species in something like the terms set out by Mayr [1, p. 120]: ‘species are groups of actually or potentially interbreeding natural populations, which are reproductively isolated from other such groups'. This ‘biological species concept′ is essentially genetic: species are kept internally cohesive by recombination and kept apart by barriers to recombination. It works well for organisms like humans that do not reproduce without sex and cannot hybridize with any other group, but many organisms, perhaps most, fall short of these ideals.

There is a prevalent view that we should not expect ‘good′ species in bacteria because even distantly related bacteria can share genes [2,3]. Nevertheless, it is undeniable that bacteria are not uniformly distributed across the potential ‘genomic space′, but form clusters. It has been argued that bacteria do have genetic processes that could provide sufficient recombination to maintain species cohesion, while minimizing homologous recombination between diverged species [4–6]. Alternatively, these clusters might reflect the underlying ecological niches provided by the environment, and this idea has been developed into the ecotype model, in which genotypic clusters map onto ecological niches and periodic selective sweeps purge genetic variation within each niche separately [7,8]. Recombination is not required in this model, and indeed could disrupt adapted ecotypes, but moderate levels can be incorporated within the model. Population genomic data for the bacterium *Vibrio cyclitrophicus* [9] and the archaeon *Sulfolobus islandicus* [10] have been interpreted in the light of these alternative genetic and ecological paradigms for speciation. In both cases, recombination played a conspicuous role in the structuring of the population, but the observed clusters mapped onto ecological differences, implicating elements of both paradigms. Genetic isolation between the clusters was incomplete and the divergence was low, suggesting that both these organisms were being observed at an early stage of the speciation process.

Here, we explore a much later stage in bacterial speciation, and observe a lack of association between genetic clusters and ecological adaptation. This leads us to question the generality of the ecotype model as a descriptor of mature species, and to propose instead that bacterial diversity is better described in terms of the concepts of genospecies and biovar. A genospecies is a discrete cluster in the sequence space of core genes, held together by recombination [4], whereas a biovar unites a set of strains that share a genetic module conferring a distinct phenotype [11].

Our study organism is the soil bacterium *Rhizobium leguminosarum*, which is well known as a nitrogen-fixing symbiont in root nodules of legume plants and has a history of molecular diversity studies stretching back thirty years [12–14]. The symbiosis is not obligate or inherited, but each nodule is established by a separate infection event from the soil, most often by descendants of a single bacterial cell. *R. leguminosarum* has distinct symbiovars [11]: symbiovar (sv.) *viciae* forms nodules only on the roots of the legume tribe Vicieae, which includes vetches, peas and lentils (*Vicia, Lathyrus, Pisum* and *Lens*), while sv. *trifolii* is confined to clovers (*Trifolium*). The ‘nodulation genes’ that define these two distinct host specificities, together with genes responsible for the process of nitrogen fixation, are normally encoded on a plasmid in this species. There are complete published genome sequences of *R. leguminosarum* sv. *viciae* strain 3841 and sv. *trifolii* strains WSM1325 and WSM2304 [15–17], and unpublished genome sequences of other strains have recently become available in the public International Nucleotide Sequence Database Collaboration (INSDC) databases. The genome of *R. leguminosarum* is large and complex, consisting of a chromosome and a variable number of low-copy-number plasmids, including two that can be called chromids [18] because they are large and carry some core genes. We sampled this species from an established plant community in Yorkshire, UK, that included hosts of the two symbiovars. We isolated 36 strains from nodules on plants of *Vicia sativa* and 36 from *Trifolium repens*, each from a separate nodule and subcultured to ensure genetic clonality. Genomic sequencing of these 72 isolates, together with phenotypic characterization, formed the basis for our observations and analyses.

The aim of the study was to determine the population structure of the core and accessory genomes within a local population of a bacterial species associated with two different hosts. We wished to assess whether the accessory genome had an overall organization or was made up of independently assorted components, and whether it reflected the phylogenetic structure of the core genome. We also wished to explore the relationship between genotype and phenotype, and the implications of this for the description of bacterial species.

## 3. Material and methods

### 3.1. Bacterial collection, DNA preparation and sequencing

Isolates were obtained at the same time, and from the same site, as the *Sinorhizobium medicae* isolates described previously [19]. Nodules were harvested on 22 March 2008 from *T. repens* and *V. sativa* plants growing on a 1 m^2^ area of roadside vegetation (grasses and herbs) located between Wentworth College and Walmgate Stray at the University of York, UK (53°56′ 44″ N, 1°03′ 35″ W). A single bacterial strain was isolated and purified from each nodule, and DNA was prepared from each isolate, as described by Bailly *et al.* [19].

Altogether, 36 isolates were obtained from *V. sativa* nodules (named VSX strains) and 36 from *T. repens* (TRX). Partial sequences of the 16S rRNA genes were obtained [20], and these confirmed that all the isolates were likely to belong to the species *R. leguminosarum* as they differed from the 16S sequences of strains 3841 and WSM1325 by at most a single nucleotide substitution.

DNA from the different strains was tagged with the Roche multiplex identifiers (MID) and sequenced on titanium plates using a GS FLX genome sequencer (Roche 454 Life Sciences, Branford, CT, USA). After initial sequence analysis, eight strains representing major clades were selected for additional sequencing using paired-end libraries with an intervening distance of approximately 4.5 kb. Coverage of each genome ranged from 6.1 to 89.6 Mb, with a median of 13.8 Mb. Details for each strain are given in the electronic supplementary material, table S1.

### 3.2. Nodulation testing

Seeds of *Vicia cracca* and *T. repens* (Emorsgate Seeds, King's Lynn, UK) were surface sterilized to remove any bacteria on the coat. They were rinsed briefly in absolute ethanol before washing in 3% sodium hypochlorite for 3–5 min. They were rinsed in seven changes of sterile de-ionized water and left to imbibe in water for 4 h, then washed in a further seven changes of water, drained and left to germinate in a covered glass beaker at 28°C. Representatives were incubated on TY agar to check for residual microbial contamination; none was found.

After germination, the seeds were placed onto prepared agar slants containing nitrogen-free minimal solution [21], each one in a separate container. *Vi. cracca* was grown in 25 ml of agar in 50 ml borosilicate glass tubes; *T. repens* was grown in 15 ml of agar in 30 ml polystyrene tubes. The surface of the slants was scratched to make a groove into which the emerging root of the seed was pushed. Bacterial suspension in liquid TY medium (0.1 ml of 1 × 10^8^ cells ml^−1^, estimated by turbidity measurement) was injected into the groove. The tubes were plugged with cotton wool until the plants had grown up to the plug, when it was replaced with plastic film with a hole in it through which the stem could grow. The plants were grown under a cycle of 16 h of light at 28°C and 8 h of darkness at 18°C and watered with nitrogen-free medium every 3 days or whenever the agar appeared dry. Four positive and four negative control replicates, with no bacteria, were also set up. The negative was watered with nitrogen-free medium and the positive was watered with the same medium with 0.05% KNO_3_ added. After 10 weeks of growth, plants were examined for the presence of pink nodules and dark green foliage, indicating effective nitrogen-fixing symbiosis.

### 3.3. Assembly and mapping of the sequence reads

Roche Newbler 2.3 assembler was run using the command line (runAssembly) option with 90% sequence identity and 40-bp minimum overlap as parameters to perform *de novo* assembly of each of the *Rhizobium* genomes. Shell scripts were written to run runAssembly on multiple datasets. GSMapper 2.3 with 90% sequence identity and 40-bp minimum overlap was used to perform reference-based assembly of each genome using 305 core genes from strain 3841 as the reference genes. These 305 genes were those shown to be common to all chromid-bearing bacteria analysed by Harrison *et al.* [18]. Shell scripts were written to run runMapper on multiple datasets. Nucleotide information of 305 core genes was extracted from every draft genome using a Perl script, and a shell script was used to merge this information with their respective genes present in fully sequenced *Rhizobium* genomes. Each of the 305 files was aligned at nucleotide level by MUSCLE [22] that was run locally on the University of York Biology Linux grid. Each alignment file was checked and gaps were added for strains that had no reads for a given gene. The final results of FASTA alignments were concatenated by strain to form a 305-gene alignment using Galaxy [23]. A 100-gene alignment was also created using only those genes that were represented in every isolate by at least one read of at least 100 nucleotides.

### 3.4. Phylogenetic analysis

Phylogenies were constructed using either neighbour-net or maximum-likelihood (ML) methods. All neighbour-nets were generated using the uncorrected p-distances function of SplitsTree v. 4.11 [24]. All maximum-likelihood analyses were performed by FastTree [25] with settings: -gamma –gtr, run locally on the University of York Biology Linux grid. For the 100-gene alignment, an ML tree was constructed using FastTree with 100 bootstrap replicates and visualized using SplitsTree. An individual ML phylogeny of each of the 100 genes was constructed using PhyML [26] with the best-fit model of nucleotide substitution calculated from ModelTest embedded in TOPALi v. 2 [27].

To compare tree topologies (e.g. single gene trees with 100-gene tree), Shimodaira-Hasegawa (SH) congruence tests implemented in the Consel package [28] were performed (*p* < 0.05: incongruent). Heatmaps to display *p*-values of SH test results were constructed with R package phylcon [29]. Pairwise homoplasy index (PHI) test computed within SplitsTree [24] was applied to each of the 100 genes with 5% significance level.

### 3.5. Average nucleotide identity and phylogenetic analysis

Average nucleotide identity (ANI) was calculated using the JSpecies package [30] using MUMmer (ANIm). The cut-off for per cent similarity between two genomes belonging to the same species is 96%, which generally gives a similar result to the DNA–DNA hybridization threshold value of 70% [31]. This method was applied to representative strains that were selected based on coverage and to include at least one member from each of the five putative genospecies (A–E) and major subclusters present in genospecies C (gsC). To assign strains from published studies to the genospecies, housekeeping gene sequences were compared by local BLASTn to a database containing all contigs from the 72 strains.

### 3.6. Analysis of population structure

Two independent runs of ClonalFrame 1.2 [32] were performed each consisting of 100 000 MCMC iterations, and the first half was discarded as burn-in. Convergence and mixing of the MCMC were found to be satisfactory by manual comparison of the runs and using Gelman & Rubin's [33] method implemented in ClonalFrame. Structure v. 2.3.4 [34] was used to identify the hypothetical ancestral populations of our isolates. Initially, ClonalFrame input (concatenated alignment of 100 core genes) was converted into Structure format using xmfa2struct (http://www.xavierdidelot.xtreemhost.com/clonalframe.htm). Four independent runs were performed for a number of populations *K* ranging from 3 to 9. For each run, 105 burn-in iterations were performed with 106 follow-on iterations. Other parameters were used as default. The optimum *K* value was evaluated by the Δ*K* method [35]. Barplots for structure results were constructed using R.

### 3.7. Presence/absence of genes

The GSMapper 2.5 software was used, with 90% sequence identity and 40-bp minimum overlap, to perform individual reference-based assembly of *R. leguminosarum* strains against the combined sequences of all Rlv 3841 replicons. Perl and R scripts were used to extract information based on Rlv 3841 genes from the output file 454RefStatus.txt, which provides information on the number of reads mapping to each reference sequence. The extracted data were converted by Perl scripts into binary presence/absence format based on at least one unique mapped read. These presence/absence matrices were displayed as heat maps using R.

### 3.8. Biolog substrate utilization assays

Each strain was assessed on duplicate Biolog GN2 plates. Bacteria were grown on TY agar plates and suspended in physiological saline, as this gave better results than the standard Biolog protocol. Then 50 μl of the inoculum (*A*_610_ = 0.1) was added to each of the 96 wells of the Biolog plate and the plates were incubated for 48 h at 28°C without shaking before reading absorbance at 590 nm on an ELISA reader (Thermomax, Thermo Scientific). Pearson correlation coefficients between utilization and the presence of genes were calculated in R using the WGCNA package [36], and genes were clustered using Euclidean distance and average linkage.

## 4. Results

### 4.1. Five genospecies in a single population

Sampling of *V. sativa* and *T. repens* root nodules in 1 m^2^ of roadside verge in Yorkshire, UK yielded 72 bacterial isolates. According to the sequences of their small subunit ribosomal RNA genes (SSU), all of the isolates might belong to the species *R. leguminosarum* since, apart from a single polymorphic nucleotide (position 1069), all SSU sequences are identical to those of the three published complete genomes from this species [15–17] and the type strain USDA2370 [37]. A recently described species, *R. laguerreae*, is closely related to *R. leguminosarum* and has the same SSU sequence [38]. There are eight fixed differences that are unique to these SSU sequences in comparison with those of the type strains of the next most closely related species *R. etli*, *R. phaseoli*, *R. pisi* and *R. fabae*.

**Figure 1.**
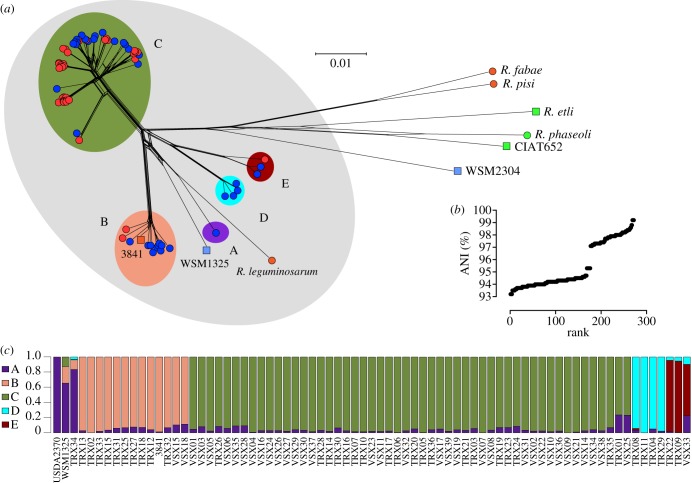
(*a*) NeighborNet phylogeny of the isolates and related sequenced bacteria based on 305 conserved genes. Symbol colour indicates symbiovar: red, *viciae*; blue, *trifolii*; green, *phaseoli*. The new isolates (unlabelled symbols) are identified in electronic supplementary material, figure S3. Named species are represented by their type strains. Round symbols indicate genome sequences obtained in this study; square symbols indicate published genome data. Background colours indicate *R. leguminosarum* as currently defined (grey), and the five genospecies A–E are identified within *R. leguminosarum* (coloured). (*b*) Ranked values of ANI for all pairwise comparisons of selected strains representing genospecies A–E, based on all shared homologous sequences (see electronic supplementary material, table S2). (*c*) Structure plot showing contribution to each strain from each of five hypothetical ancestral populations.

A phylogeny based on the concatenated sequences of 305 conserved core genes confirms that the isolates are indeed more similar to each other than to any of the related species (figure 1). However, it is striking that the isolates fall into five discrete clusters. ANI [39] calculated for representative isolates is consistently above 96% (96.3–100%) for pairs in the same cluster and below 95% (92.4–94.6%) for pairs in different clusters (figure 1; electronic supplementary material, table S2). An ANI value of 95% has been shown to correspond to a DNA–DNA hybridization value of 70% [40], and thus to the level of divergence traditionally used to separate bacterial species. The five clusters A–E (figure 1) are sufficiently diverged, therefore, to be recognized as separate species. We may call them genospecies, and they are cryptic species unless we find clear phenotypic characters to distinguish them. The single variable nucleotide in the SSU sequence (position 1069) reflects the phylogeny, being T in genospecies A (gsA) and in gsB, C in the majority of gsC, though A in one subclade, A in gsD and C in gsE. We also used ANI to determine that USDA2370^T^, the type strain of the species *R. leguminosarum*, belongs to gsA (electronic supplementary material, table S2). Based on their published complete genome sequences [15,17], *R. leguminosarum* strain 3841 falls within gsB, while WSM1325 has the highest ANI with TRX34, representing gsA. This latter value is slightly below 95%, though, so the affiliation of WSM 1325 to gsA is ambiguous, in agreement with its position in the phylogenetic network shown in figure 1.

It is worth noting that the diversity represented here in a single population of *R. leguminosarum* is at least 10 times higher than that found among isolates of *S. medicae* isolated at the same time and from the same site [19]. Even the diversity within each genospecies is higher than that in the whole *S. medicae* population. Clearly, bacterial populations can vary greatly in their level of genetic diversity. Another population genomic analysis of *S. medicae* and *Sinorhizobium meliloti* also found relatively low levels of diversity, suggesting that this may be characteristic of these species [41].

The high level of divergence among the five genospecies implies that they have been distinct for a long time and did not originate in this particular location. Indeed, there is evidence that they have had separate identities long enough to have spread around the world. A number of draft genomes of *R. leguminosarum* strains have recently been submitted to INSDC, and these were included in an additional ANI analysis (electronic supplementary material, table S3). Besides strain 3841 (from Norfolk, UK), VF39 (Bielefeld, Germany) and WSM1481 (Greece) are unambiguously included in gsB. Strains TA1 (Tasmania, Australia) and GB30 (Janow, Poland) are in gsC, as are Vh3, Vc2 and Ps8, three strains isolated from soil collected about 250 m from the present study site [42]. A group of strains that share very high ANI (99–100%) but have disparate origins, 4292 (Norfolk, UK), CC283b (Russia), UPM1137 (Italy) and 128C53 (a US inoculant), belong in gsE, as they are close to TRX09. CC278f (Colorado, USA) is close enough to TRX11 (95.7% ANI) to be considered a rather diverged member of gsD. SRDI943 (Victoria, Australia) has 98.7% ANI with WSM1325 (Serifos, Greece) which, as already pointed out, is close to gsA. Thus, strains close to all five of the genospecies have been found in disparate parts of the world. Other studies of *R. leguminosarum* diversity have used sequences of two or three core protein-coding genes. While this is clearly not as reliable as whole genome ANI, these gene sequences can indicate whether strains might belong to the genospecies we have defined. Santillana *et al.* [43] isolated strains in Peru, and we determined, on the basis of their *recA* and *atpD* sequences, that strains PEVF03, PEVF09 and PEVF10 are gsB, while PEVF01 and PEVF02 are gsE. On the other hand, PEVF05 and PEVF08 cannot be allocated to the genospecies we have defined. Similarly, Tian *et al.* [44] placed some Chinese isolates together with strain 3841 in a tight cluster that they called *Rlv-VII*, which is equivalent to our gsB. They also defined a number of other clusters that contained only Chinese strains, and these are distinct from our genospecies. Strains belonging to one of these clusters, *Rlv-V*, were also found in the Spiti valley in Himachal Pradesh, India, while the adjacent Lahaul valley had strains very similar to the type strain USDA2370^T^, which is gsA [45]. Overall, we can conclude that our genospecies are globally distributed and that there are other potential genospecies within *R. leguminosarum sensu lato* that are also widely distributed.

### 4.2. Restricted recombination of a large part of the genome

The five genospecies are separated by long, strongly supported branches in the phylogeny (figure 1), implying that the 305 core genes used to construct this phylogeny are rarely recombining between genospecies. This was investigated in detail for a subset of 100 of these genes for which sufficient coverage was available for all 72 isolates; the published genomes of 3841 and WSM1325, and our unpublished data for the type strain USDA2370 were also included. Half of these core genes (50/100) had ML phylogenies that were significantly incompatible with the topology of the ML phylogeny derived from the concatenated alignment of all 100 genes (Shimodaira-Hasegawa test). Furthermore, analysis of recombination using ClonalFrame [32] indicated a high rate of recombination among the 75 strains, with *ρ*/*θ* = 1.32 (the ratio of recombination to mutation events) and *r/m* = 5.92 (the probability that a site is affected by recombination rather than mutation). Values of *r/m* > 2 are considered high [46]. These analyses indicate substantial levels of recombination across the 75 strains, but this includes events occurring within genospecies as well as between them. We repeated the ClonalFrame analysis separately for each of the two best represented genospecies, demonstrating high recombination within gsC (*ρ*/*θ* = 0.79, *r/m* = 4.29, *n* = 52 strains) and extremely high recombination within gsB (*ρ*/*θ* = 26.59, *r/m* = 102.93, *n* = 13 strains). This latter observation is consistent with the lack of resolution in the star-like phylogeny of gsB (figure 1). These high *r/m* ratios imply that recombination provides an effective cohesive force within these genospecies, as it is sufficiently frequent to prevent divergence by neutral drift.

Surprisingly, the strong restriction on recombination between genospecies is not confined to the core genes on the chromosome, but extends to many genes on the chromids and larger plasmids. Phylogenetic analysis based on all the sequences in the 72 isolates that map to the genome of Rlv3841 (electronic supplementary material, figure S1) yields strikingly similar relationships for the chromosome, the two chromids (pRL12 and pRL11) and even the next two plasmids in size (pRL10 and pRL9). Only the two smallest plasmids (pRL8 and pRL7) break this pattern, but the less resolved networks displayed by these replicons are, at least in part, a consequence of the fact that very few of the genes that they carry are widespread among the 72 isolates.

Although the phylogenies based on genes associated with the large plasmids are very similar to that based on the chromosome, there are some differences that demonstrate the occasional transfer of large plasmids within gsC. For example, strain VSX32 groups with VSX23 and TRX20 in the chromosomal phylogeny and that based on pRL12, but is in the very tight VSX04 clade based on genes found on pRL10 and pRL9, and on the edge of it for pRL11 genes.

### 4.3. Accessory genes move within and between genospecies

Accessory genes differ from core genes in two ways: they are not necessarily present in all strains, and they may have independent phylogenies that differ from that of the core genome [15,47]. These are the expected consequences of their mode of inheritance. Accessory genes are carried by mobile elements—plasmids, islands, transposons, phages—that move horizontally from strain to strain, and accessory genes do not depend on homologous recombination for maintenance in the recipient.

In rhizobia, the best known accessory genes are those involved in nodulation of the host plant (*nod*) and nitrogen fixation (*nif* and *fix*) in the nodules, so we can readily use these as an example to explore the behaviour of accessory genes. Our sample of strains was selected from root nodules, so we expect all isolates to carry these genes for symbiosis. The exception that proves the rule is strain VSX18. This lacks the *nifHDK* genes for nitrogenase and is the only isolate that did not form nitrogen-fixing nodules when tested on its original host species. It is possible that these genes were lost in culture after isolation.

**Figure 2.**
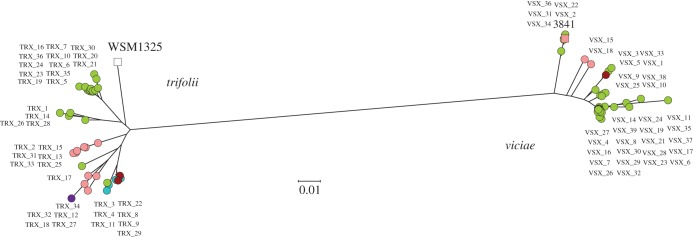
Phylogeny of the concatenated nodulation genes (*nodABCDEFIJLMN*) for each of the two host range types, symbiovars *viciae* and *trifolii*. The genospecies of each isolate is identified by its colour (see figure 1).

It is these symbiosis-related genes that define the distinct host ranges of symbiovars *viciae* and *trifolii*. The first striking observation is that the two biovars are intermingled in the different genospecies (figure 1). This confirms an observation made many years ago, that both symbiovars occurred in a similar range of distinct genetic backgrounds [12]. In another early study, RFLP variants of the sv. *viciae* symbiosis gene region showed not only an association with background genotypes but also some indication of transfer between them [14]. With the benefit of sequence information, we can take this further and explore the phylogeny of the *nod* genes within each symbiovar (figure 2). Each clade in the *nod* gene tree is predominantly associated with a particular genospecies, and often with a particular group of strains within a genospecies, but there are multiple examples of distantly related strains sharing closely related *nod* genes, which must indicate relatively recent horizontal transfer of these genes. For example, VSX33 (gsE) has *nod* genes similar to those of VSX1, VSX3 and VSX5, a close-knit group in gsC. The *nod* genes of TRX03 (gsC) are close to those found in gsD and gsE, while those of TRX01, TRX14 and TRX26 are similar although these strains are dispersed in different clades of gsC. Furthermore, there are five strains that have *nod* genes very similar to those of the reference strain 3841, but these are in gsC whereas 3841 (isolated more than 30 years earlier, 200 km away in Norfolk) is in gsB. Close relatives of these five strains have *nod* genes belonging to two other distinct clades within sv. *viciae*. Closely related strains may even belong to different symbiovars, e.g. VSX25 and TRX01, VSX32 and TRX20. This picture, in which strains with closely similar core genomes have very different *nod* genes, while genetically distant strains share similar *nod* genes, demonstrates that there have been repeated transfers of the symbiosis gene cluster between and within the genospecies that make up *R. leguminosarum*.

**Figure 3.**
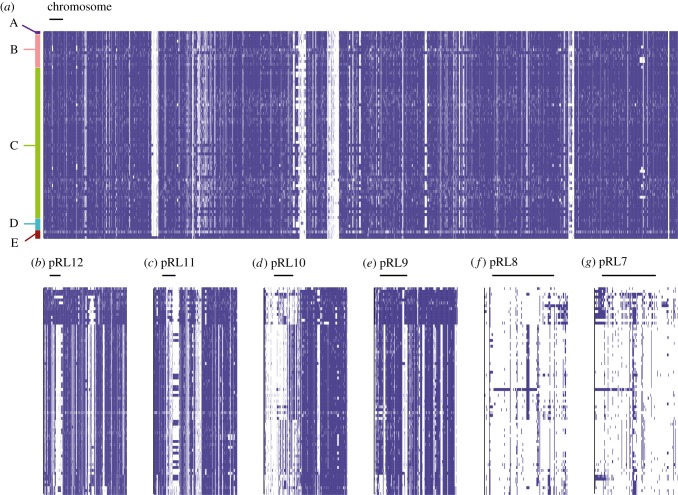
Distribution of genes across the population (blue, present; white, absent), arranged horizontally in their order on the replicons of strain 3841. Scale bars indicate 100 genes. The 72 strains are grouped vertically by genospecies (A–E).

Is this typical of accessory genes? To provide a broad overview of the accessory genome, each gene in the reference strain 3841 was used as a BLASTn query to search for close homologues in the sequence data for all strains (figure 3). Core genes can be identified as those which are found in all strains (or nearly all, as a few will be missed because of the limited sequencing depth). Predictably, most chromosomal genes meet this definition of core, as do a substantial fraction on the chromids and larger plasmids, while the two smallest plasmids have few core genes. A close examination of the data underlying figure 3 reveals that most isolates have a unique combination of genes. Closely related strains can nearly always be distinguished by at least one cluster of five or more genes that are adjacent in the 3841 genome. The only exceptions are the two strains VSX22 and VSX31, and the three strains VSX24, VSX27 and VSX29. Each of these two groups is within one of the tight clusters within gsC in the core gene tree (figure 1), so might represent recent clonal sibs.

### 4.4. Each genospecies has some unique accessory genes

While many genes have distributions that cut across the genospecies boundaries, there are some that appear to be genospecies-specific. In the analysis presented in figure 3, this is only really evident for gsB, because the focus is on genes present in the reference strain 3841, and this is a member of gsB. The figure shows some genomic islands, especially on pRL9, that are found only in gsB strains. Potentially, these might confer specific phenotypes that could be used to characterize and identify this genospecies, but unfortunately their specific functions, like those of most of the accessory genome, are unknown at present. A list of the genes found exclusively in gsB, with their annotation in the 3841 genome, is provided in the electronic supplementary material, table S4.

**Figure 4.**
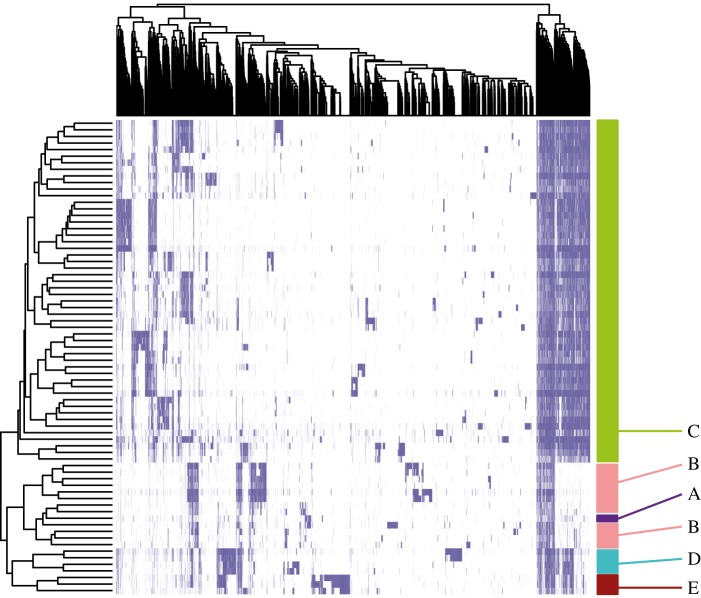
Distribution of genes that are found in the Wentworth population but are absent from strain 3841. Both strains (rows) and genes (columns) are sorted by their presence/absence pattern (blue, present; white, absent).

By assembling the reads that did not map to the reference genome of strain 3841, we obtained 8802 contigs with a total size of 11 250 877 bp. These contigs were automatically annotated by the RAST server [48], resulting in 13 252 predicted coding sequences (CDS). This represents the pool of accessory genes available at this location but not present in the reference strain. It is certainly an underestimate of the true number because the low sequencing coverage of some isolates will lead to genes being missed. The distribution of these CDS across the 72 strains is shown in figure 4, in which the strains have been sorted by the similarity of their gene content and the genes have been sorted by the similarity of their distribution across strains. The strains are sorted almost perfectly into genospecies, indicating that strains within a genospecies have more similar gene content, and there are evident clusters of genes that are characteristic of each genospecies. Potentially, these genes could confer distinct phenotypes on each genospecies, but elucidating all of these would be an immense task. In a genomic hybridization study, Lasalle *et al.* [49] similarly found genes specific to a genospecies (genomovar) of *Agrobacterium*, and were able to confirm functions experimentally for a few of them.

### 4.5. Metabolic diversity and the genes responsible for it

**Figure 5.**
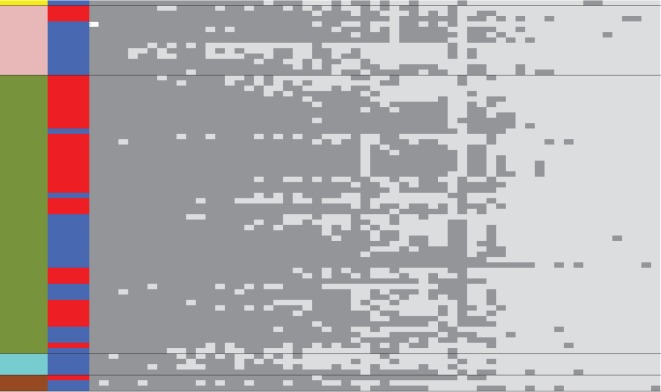
Utilization of carbon substrates by the 72 *R. leguminosarum* strains plus the reference strain 3841 (rows), determined using the Biolog GN microplate. Colour at left indicates the genospecies (gsA–gsE, figure 1); the second column indicates symbiovar (red, *viciae*; blue, *trifolii*). The pattern of utilization (dark grey) or non-utilization (light grey) is shown for the 59 substrates (columns) that were used by some but not all strains. See the electronic supplementary material, table S6 for the details of strain and substrate identity.

Each of the 72 isolates was screened for the ability to oxidize a panel of 95 carbon sources using the Biolog GN microplate (figure 5; electronic supplementary material, table S6). All isolates could use 23 of the substrates, while 13 compounds were not used by any of the isolates. The isolates were diverse in their ability to use the remaining 59 compounds. Indeed, every strain had a unique metabolic pattern except for two, VSX16 and VSX27, that are also extremely similar in phylogeny and gene content. It is worth noting that VSX22 and VSX31, which could not be distinguished clearly by gene content (§4.3), nevertheless had very distinct metabolic phenotypes: VSX31 used an additional nine substrates that were not used by VSX22. The other cluster of three isolates that shared gene content profiles, VSX24, VSX27 and VSX29, were also metabolically diverse, using 57, 65 and 61 substrates, respectively. Of course, some metabolic differences could reflect allelic differences rather than the presence or absence of genes.

Patterns of utilization are not strongly associated with either the genospecies or the symbiovar. No substrate was used exclusively by a single genospecies or symbiovar, unless it was rarely used at all (by no more than three isolates). This suggests that the genetic determinants underlying the majority of metabolic differences were distributed in a pattern that did not reflect the relationships of either the core genomes or the nodulation genes.

Candidate genes conferring a metabolic capability may be indicated by a strong positive correlation between substrate utilization and presence of the gene. In an effort to identify some of the causal genes, the correlation between substrate utilization and gene presence was calculated for all substrates that varied across the isolates. The correlations for genes on plasmid pRL10 are illustrated in the electronic supplementary material, figure S2. The strongest signal was for utilization of γ-hydroxybutyrate and a cluster of six genes, pRL100133 to pRL100138 (protein accessions YP_770415 to YP_770420). These genes (electronic supplementary material, table S5) are homologues (60–90% amino acid identity) of the *attJ*, *attk*, *attL*, *attM* genes of *Agrobacterium tumefaciens* [50] plus *metX* (homoserine *O*-acetyl transferase) and a gene encoding a MerR-family transcriptional regulator. Carlier *et al.* [50] demonstrated that *attJKLM* allowed the conversion of γ-butyrolactone to succinate via γ-hydroxybutyrate. We confirmed that these genes, played a similar role in *R. leguminosarum* 3841 by mutational knock-out of pRL100135 (*attL*), which abolished the ability to grow on γ-hydroxybutyrate (G. Lad 2013, unpublished data). The other two genes have no close homologues in *A. tumefaciens* C58 and should not be necessary for the assimilation of γ-hydroxybutyrate, but may be involved in related metabolism. There are three further genes in another location on pRL10 that also show a high correlation with γ-hydroxybutyrate utilization: pRL100103 encodes an alcohol dehydrogenase that is a more distant homologue of AttL (51% amino acid identity), while pRL100104 and pRL100105 encode subunits of a possible polyhydroxybutyrate synthase. Although these genes are not adjacent on pRL10, their distribution among our isolates suggests that they may be transferred as a group. γ-Hydroxybutyrate is used by 34 of the 72 isolates, including some members of genospecies B, C, D and E, and both symbiovars, although utilization is significantly more frequent in sv. *trifolii* (25/36) than in sv. *viciae* (9/36; *χ*^2^ = 14.3, *p* < 0.001). The distribution of the *att* gene cluster is similar, except that six isolates (TRX32 and VSX18 in gsB, VSX16, VSX26, VSX27, VSX37 in gsC) that lack any genes of the *att* cluster are nevertheless able to use γ-hydroxybutyrate, implying that they possess an alternative pathway for which the genes are currently unknown. This example confirms that the observed metabolic diversity in our *R. leguminosarum* population can (at least in principle) be related to the underlying distribution of genes.

### 4.6. Metabolic characteristics are not good taxonomic markers

The guidelines for the description of new bacterial species currently require the inclusion of phenotypic data, especially discriminating markers that can distinguish a particular species from others [51,52]. Accordingly, the published descriptions of *R. leguminosarum* and related species include lists of substrates that can, or cannot, be used for growth [37]. We tested the utility of this information using the Biolog results that we obtained for our 72 isolates, all of which we have shown (figure 1) to belong to the species *R. leguminosarum*, as currently defined, rather than to the related species *R. pisi* or *R. phaseoli*.

The published utilization patterns [37] actually have limited power to distinguish among these closely related species, since the three species have the same pattern for most substrates (table 1). Only l-alanine (used by *R. leguminosarum* and *R. pisi* but not *R. phaseoli*) and l-serine (used only by *R. pisi*) promise to provide unambiguous species identifications. Our Biolog data show that this is illusory, however (table 1). Only 85% of our *R. leguminosarum* isolates could grow on l-alanine, while 53% grew on l-serine. The strains also showed varying responses to the majority of the other substrates tested. It seems clear that the supposedly diagnostic differences in phenotype were based on a limited sampling of the diversity within each species. Unfortunately, most published species descriptions are based on a similarly small number of strains. Our data suggest that substrate utilization patterns can vary greatly within a single bacterial species. Indeed, even the five cryptic genospecies that we have identified within the recognized species *R. leguminosarum* do not have distinct and consistent differences in their substrate utilization (figure 5).

**Table 1.** The observed utilization of carbon substrates by 72 isolates of *R. leguminosarum* in this study, and the diagnostic utilization according to the species descriptions of *R. leguminosarum*, *R. phaseoli* and *R. pisi* [37]. Usage by the 72 isolates was determined using Biolog GN plates (figure 5; electronic supplementary material, table S6). According to the species descriptions, substrates should be invariably used (100) or never used (0) by a species, while V indicates variable usage [37]. Blanks indicate data not available.

substrate	observed	species description		
	% of strains utilizing	*R. leguminosarum*	*R. phaseoli*	*R. pisi*
glucose	100	100	100	100
l-arabinose	100	100	100	100
fructose	100	100	100	100
galactose	96	100	100	100
l-rhamnose	99	100	100	100
xylose		100	100	100
melibiose		100	100	100
cellobiose	100	100	100	100
mannose	100	100	100	100
mannitol	97	100	100	100
sorbitol	100	100	100	100
inositol	96	100	100	100
xylitol	93	100	100	100
*N*-acetyl-glucosamine	54	100	100	100
maltose	100	100	100	100
raffinose	88	100	100	100
sucrose	100	100	100	100
trehalose	100	100	100	100
salicin		100	100	100
l-alanine	85	100	0	100
l-histidine	100	100	100	100
aspartate	57	100	100	100
glutamate	72	100	100	100
betaine		100	100	100
sarcosine		100	100	100
erythritol		V	0	0
l-arginine		V	0	0
l-malate		V		100
gluconate	89	V	100	100
l-sorbose		0	0	0
melezitose		0	0	0
caproate		0	0	0
adipate		0	0	0
citrate	7	0	0	0
pyruvate		0	V	100
propionate	1	0	0	0
phenylacetate		0	0	0
l-serine	53	0	0	100
l-lysine		0	0	0
l-valine		0	0	0

## 5. Discussion

### 5.1. The origin and maintenance of diversity

We have observed a local population of *R. leguminosarum* made up of at least five distinct genospecies that maintain their identity over wide geographical distributions. The strains in each genospecies are heterogeneous in their content of accessory genes and in their phenotypes, most conspicuously in their symbiotic host range determinants. What are the processes that created such a population and currently maintain it?

Concepts of bacterial populations that are based on clonality have a long history and have been very influential. Muller described how the spread of new mutations in an asexual population would crowd out variants in other clonal lineages [53], and this consideration of clonal competition within a species was echoed by Gause's principle of competitive exclusion between species in the same niche [54]. These ideas suggest that distinct lineages will not coexist indefinitely unless they have ecological differences that give them distinct niches. Furthermore, when a superior variant arises, it will spread through the population, sweeping away the accumulated genetic variation—a process known as periodic selection that can readily be demonstrated in simple laboratory cultures [55]. Periodic selection can maintain genetic cohesion of strains that share an ecological niche, and the potential of this mechanism to explain the population structure of bacteria has been explored extensively, especially by Cohan and co-workers [7,8,56,57]. It provides a plausible mechanism for the early divergence of incipient bacterial species, creating clusters in genotype space that correspond to ecotypes, i.e. sets of ecologically equivalent strains. The clusters of strains described in the bacterium *Vibrio cyclitrophicus* [9] and the archaeon *Sulfolobus islandicus* [10], which show ecological coherence and low levels of genetic divergence, are possibly examples of this. In the case of our *R. leguminosarum* population, this level of genetic divergence (less than 0.004 substitutions per site) corresponds to the differences among closely related strains within a single genospecies (figure 1). It is below this level of genetic divergence that we could expect to see ecological coherence, but the overall diversity of the population is so high that our sample of 72 isolates does not include any clear example of the same ecotype being sampled twice. Every isolate is unique in gene content (figure 3) or in substrate utilization (figure 5), or both. A similar situation was recently reported in *Bacillus subtilis* by Kopac *et al.* [56], who demonstrated that every one of a number of closely related isolates had a unique combination of ecological properties, so constituted a unique ecotype. They described these as arising through a ‘nanoniche′ model of bacterial speciation in which ecotypes flit briefly into existence as a result of minor genetic change, but soon disappear as they are quashed by competition from others with overlapping niches [56]. These ecotypes are not species in any conventional sense because they do not have the long-term separateness and recognizability that most species concepts require. In our view, they are more akin to individuals in a sexual population, each to a greater or lesser degree unique.

It is certainly striking that our *R. leguminosarum* population is so far from being clonal. It seems that, by the time the descendants of a root nodule occupant have moved away and founded their own nodules, each has gained or lost genes and become genetically unique. In such a situation, the simple clonality-dependent mechanisms of periodic selection and competitive exclusion are unlikely to have much traction. Weidenbeck & Cohan [57] considered ways in which gene transfer might be incorporated into ecotype-based models of bacterial diversification. The model they called ‘recurrent niche invasion′ comes closest to describing the situation that we observe. This posits that niche is determined by plasmids that can be gained or lost by lineages. Periodic selection events affect all individuals that are currently adapted to a particular niche, regardless of the lineage they belong to. The result is that periodic selection promotes the cohesion of the niche-determining genes, but not of the genetic backgrounds that carry them. The symbiosis genes of *R. leguminosarum* appear to meet this definition of niche-determining genes. A mutation in these genes that increases competitiveness for nodulation of clover, for example, may sweep through symbiovar *trifolii*, eliminating less-competitive variants of these genes. In the absence of gene transfer, the core genetic background that carried the successful genes might also hitch-hike to high frequency, but plasmid transfer will eventually move the successful nodulation genes into other background genotypes. In any case, many strains in the population do not carry *trifolii* genes and are not competing for the clover nodule niche: strains of symbiovar *viciae* will be unaffected by selection for improved nodulation of clover. If this kind of cohesive selection is particularly important, one would expect that the symbiosis genes within a symbiovar would show lower levels of polymorphism than other regions of the genome. In an earlier study [19], we demonstrated that this was true of the *S. medicae* population at our study site. However, the nodulation genes in each of the *R. leguminosarum* symbiovars show similar levels of divergence (up to 6%, figure 2) to those seen for core genes across the whole set of genospecies (figure 1), so there is no evidence for a recent periodic selection event purging variation within either the *trifolii* or the *viciae* symbiovar.

Vetches and clovers are native in many parts of the world, so it is only to be expected that their bacterial symbionts, belonging to *R. leguminosarum* and related species, are also widespread. What is more surprising is that several distinct genospecies coexist at one site, and the same genospecies are found in other regions where the local conditions must be substantially different. Genospecies A (or close relatives) has also been found in Greece, Australia, India and USA; gsB in Germany, Greece, China and Peru; gsC in Poland and Australia; gsD in USA; gsE in Russia, Italy, USA and Peru (§4.1). These results must be interpreted with some caution, because rhizobia are important for agricultural crops and some have certainly been moved around the world deliberately as inoculants as well as accidentally along with crop seeds [43]. Nevertheless, many of these reports are not from crops, but from native wild legumes in natural habitats, and all of them indicate the ability of these genospecies to succeed in a wide range of conditions. We have observed a very large pool of accessory genes in our population, several-fold higher than the core gene pool. There are several hundred genes that, in our population, are confined to each of the five genospecies, suggesting that each genospecies has potential adaptations that make it distinct from the others (figure 4). It remains to be determined whether these genes remain associated with the same genospecies wherever they are found in the world. It is conceivable that these genes include some that were important in the origin of the genospecies because they conferred adaptations that gave the genospecies access to a new niche, but this origin was a long time ago and both core and accessory genomes have changed extensively since, so it not straightforward to reconstruct those distant events, or even to know whether the initial separation between genospecies was the result of ecological [7,8] or genetic [6] processes.

There are several hundred genes that are found in virtually all of the strains in our population (figure 4), regardless of genospecies or symbiovar, but are absent in the reference strain 3841, which was isolated 200 km away in Norfolk, UK. We can speculate that these genes include some that confer adaptations that are important in the specific conditions of our sampling site. There is evidence from studies of other rhizobia that resident strains are better adapted to their local environment than incoming strains are. When a *Mesorhizobium loti* inoculant was introduced in New Zealand to provide a symbiont for a crop of non-native *Lotus corniculatus*, the original inoculant was supplanted after a few years by a diversity of local *Mesorhizobium* strains that had all acquired the host-specific nodulation genes introduced by the inoculant [58]. A similar story unfolded after inoculation of *Biserrula pelecinus* in Australia [59]. These cases confirm that accessory genes can spread rapidly through a bacterial population if they confer a significant advantage under the prevailing conditions. We can expect that, although recognizable genospecies within *R. leguminosarum* may be found across the world, these will have access to a pool of adaptive ‘local genes' that will differ from site to site.

### 5.2. The failure of polyphasic taxonomy

In a discussion paper published more than a quarter of a century ago, an ad hoc committee of taxonomists had the prescience to declare ‘There was general agreement that the complete deoxyribonucleic acid (DNA) sequence would be the reference standard to determine phylogeny and that phylogeny should determine taxonomy. Furthermore, nomenclature should agree with (and reflect) genomic information.′ [60, p. 463]. At that time, the ‘best applicable procedure′ for comparing genome sequences was DNA–DNA reassociation. This is not a practicable method for diagnostic identification of bacteria, so the committee recommended that a distinctive phenotypic property should be identified before describing a new species. Despite this early assertion of the primacy of genomes in taxonomy, the *de facto* standard continued to be polyphasic taxonomy, which dates back even earlier [61] and aims at ‘the integration of different kinds of data and information (phenotypic, genotypic, and phylogenetic)’ [62, p. 408]. To this day, journal editors and reviewers continue to expect the description of a new bacterial species to be supported by a range of phenotypic as well as genotypic data. Even Vandamme, a leading proponent of the polyphasic approach, now concedes that this imposes demands that are ‘counterproductive′ and have held back the description of new taxa [63]. The solution that Vandamme and Peeters propose is to require just ‘a full genome sequence and a minimal description of phenotypic characteristics' [63, p. 57] for each new species. While we agree with the diagnosis of the ailment, our findings suggest that this will not be an effective remedy. A single genome sequence cannot provide an adequate description of a species, as it gives no indication of the diversity encompassed by the species (§4.1). Capturing this diversity requires multiple genomes spanning the range of genetic variation across the species or, failing this, a single genome that is complemented by data on polymorphism of a handful of core genes sequenced in multiple strains. Furthermore, our data indicate that simple phenotypes are unlikely to provide reliable diagnostic tests for a species, as commonly used features may not be consistent when enough strains are examined (§4.6). There may be sets of genes that are unique to each species (§4.4), and we can surmise that these provide species-specific phenotypes, but these phenotypes have not, in general, been elucidated. In any case, it is likely that many of them are complex and not amenable to simple diagnostic tests and that strains will eventually be found that lack the phenotype while still belonging, genomically, to the species. Ormeño-Orrillo and Martínez-Romero [64] have made a similar argument, documenting some of the complex substrates that rhizobia have been shown to use, and pointing out that such phenotypic properties are easily lost or gained and will differ within a species. We agree completely with their conclusion that ‘long lists of substrates used by rhizobia are published in descriptions of novel species and they have very little practical use, thus being a waste of time and effort′ [64, p. 146]. It is time to implement the vision of the ad hoc committee [60] and let the sequence of core genes determine phylogeny, and phylogeny determine taxonomy, without confusing the issue with fickle phenotypes.

### 5.3. A general framework for bacterial diversity

The paradigm that best describes this bacterial population is one of *genospecies* and *biovar*. The genospecies describes a discrete cluster of strains, defined by core gene sequences, that provides a stable basis for taxonomy. The biovar describes a significant phenotype conferred by a group of genes that are commonly transferred together between strains and, potentially, between species. Such transfer may lead to a ‘disconnection′ between taxonomic and functional composition in bacterial communities, as noted by Burke *et al.* [65].

This paradigm is widely applicable and may often provide a clearer description of the situation than the conventional terminology. The complex suites of characters required for interactions with a eukaryote host provide numerous examples. For example, the pathogen that causes anthrax is commonly called *Bacillus anthracis*, and the source of insecticidal BT toxin is called *B. thuringiensis*, but bacteriologists have recognized for decades that their pathogenic properties are almost the only consistent feature that separates them from *B. cereus* [66] and that ‘these species are not strictly based on genomic divergence … but rather on subjective consideration of practical usefulness’ [67, p. 851]. While the *anthracis* phenotype has typically been associated with a restricted range of chromosomal types, the genes responsible are plasmid-encoded and can be found in other *B. cereus* backgrounds, where they can function and cause anthrax [68,69]. The *thuringiensis* plasmids, on the other hand, are found across a wide range of *B. cereus* core genomic backgrounds [70]. The situation here is directly comparable with that in *Rhizobium*. It is not appropriate to describe *anthracis* and *thuringiensis* as subspecies of *B. cereus*, because that would imply that their core genomic backgrounds were distinct, though not diverged enough for full species. Essentially, we have here *B. cereus* biovar *anthracis* and biovar *thuringiensis*, where the distinctive phenotype of each biovar is plasmid-borne and transmissible across a range of core genomic backgrounds. Since the phenotype is pathogenesis, we might be more specific and call them ‘pathovars’, although this term is more frequently used of plant pathogens.

Agrobacteria are plant pathogens with a distinctive mode of operation: they conjugate with plant cells and transfer genes from a plasmid to the plant nucleus. The transferred DNA induces either crown galls (Ti plasmid) or root proliferation (Ri plasmid). We note that the pathogenic characteristics are, once again, conferred by plasmid-borne genes. *Agrobacterium* and *Rhizobium* are closely related genera. The majority of tumour- or root-inducing isolates fall within the *Agrobacterium* clade, but some are clearly in *Rhizobium* [71] or other related genera of the Alphaproteobacteria [72]. Conversely, root-nodulating bacteria (rhizobia) are sometimes found within the phylogenetic genus *Agrobacterium* [73]. In these cases it is clear that the taxonomy of the bacterium, even at the genus level, is not a good guide to its salient phenotype, which is conferred by potentially mobile plasmid-borne genes. If our classification is to reflect biological reality, we cannot include the phenotype in the definition of genus or species, but must add it as a biovar designation—the nomenclatural equivalent of a plasmid that is potentially shared among species. Of course, it is in the nature of the accessory genome that sets of functions can come together in different combinations, and it is possible to envisage a bacterium that has the ability both to form root nodules and to cause a proliferative disorder, i.e. to be simultaneously a rhizobium and an agrobacterium (using these terms to describe phenotype, not taxonomy). Indeed, such bacteria have been reported [74], demonstrating that biovars, like plasmids, are not necessarily mutually exclusive.

Bacterial species are traditionally defined by ‘polyphasic taxonomy′, requiring both phylogenetic coherence and distinctive phenotypic traits, but this does not map well onto the biology of bacteria. A consistent taxonomy of bacteria cannot combine both genomic and phenotypic criteria, and we argue that bacterial systematics should, in future, be based on core gene relationships without requiring that a species should necessarily be phenotypically homogeneous. Some major suites of correlated adaptive traits, such as symbiotic host range or pathogenicity, merit recognition as ‘biovars'. This approach is widely applicable and is consistent with our current understanding of the diversity and evolution of bacteria, which has emerged in the past decade with the abundant availability of genome sequences.

## Supplementary Material

Bacterial genopecies supplementary information

## Data accessibility

Data are available from the Sequence Read Archive of the International Nucleotide Sequence Database Collaboration as study accession PRJEB7987.
