# Supplementary material for: Bacterial genospecies that are not ecologically coherent: population genomics of Rhizobium leguminosarum
Source: Open Biol. 2015 Jan 14;5(1):140133. doi: 10.1098/rsob.140133 (PMC4313370; doi:10.1098/rsob.140133)
Supplement: Bacterial genopecies supplementary information [file rsob140133supp1.docx]

**Electronic Supplementary Material for:**

**Bacterial genospecies that are not ecologically coherent: population genomics of *Rhizobium leguminosarum***

Nitin Kumar, Ganesh Lad, Elisa Giuntini, Maria E Kaye, Piyachat Udomwong, N Jannah Shamsani, J Peter W Young* and Xavier Bailly

^*^ [peter.young@york.ac.uk](mailto:peter.young@york.ac.uk)

Table S1. Sequencing coverage for each isolate and for type strains that were also sequenced.

| **Strain** | **Coverage (Mb)** |
| --- | --- |
| VSX_1 | 12.9 |
| VSX_2 | 17.4 |
| VSX_3 | 6.2 |
| VSX_4 | 26.8 |
| VSX_5 | 43.2 |
| VSX_6 | 22.9 |
| VSX_7 | 54.6 |
| VSX_8 | 11.1 |
| VSX_9 | 53.7 |
| VSX_10 | 19.5 |
| VSX_11 | 24.8 |
| VSX_14 | 14.1 |
| VSX_15 | 11.2 |
| VSX_16 | 13.8 |
| VSX_17 | 13.9 |
| VSX_18 | 9.6 |
| VSX_19 | 21.9 |
| VSX_21 | 21.6 |
| VSX_22 | 18.6 |
| VSX_23 | 13.8 |
| VSX_24 | 12.0 |
| VSX_25 | 18.9 |
| VSX_26 | 13.8 |
| VSX_27 | 10.7 |
| VSX_28 | 8.7 |
| VSX_29 | 7.1 |
| VSX_30 | 9.7 |
| VSX_31 | 9.3 |
| VSX_32 | 11.3 |
| VSX_33 | 7.1 |
| VSX_34 | 8.6 |
| VSX_35 | 8.7 |
| VSX_36 | 7.7 |
| VSX_37 | 9.9 |
| VSX_38 | 9.7 |
| VSX_39 | 11.8 |
| TRX_1 | 20.8 |
| TRX_2 | 48.1 |
| TRX_3 | 52.1 |
| TRX_4 | 17.9 |
| TRX_5 | 25.8 |
| TRX_6 | 89.6 |
| TRX_7 | 19.5 |
| TRX_8 | 23.8 |
| TRX_9 | 24.5 |
| TRX_10 | 22.7 |
| TRX_11 | 50.0 |
| TRX_12 | 18.2 |
| TRX_13 | 15.6 |
| TRX_14 | 25.6 |
| TRX_15 | 22.6 |
| TRX_16 | 12.8 |
| TRX_17 | 23.0 |
| TRX_18 | 21.2 |
| TRX_19 | 16.1 |
| TRX_20 | 9.7 |
| TRX_21 | 25.9 |
| TRX_22 | 19.8 |
| TRX_23 | 10.6 |
| TRX_24 | 7.9 |
| TRX_25 | 9.2 |
| TRX_26 | 7.6 |
| TRX_27 | 7.7 |
| TRX_28 | 9.3 |
| TRX_29 | 11.0 |
| TRX_30 | 9.4 |
| TRX_31 | 6.1 |
| TRX_32 | 7.7 |
| TRX_33 | 11.1 |
| TRX_34 | 6.5 |
| TRX_35 | 10.9 |
| TRX_36 | 10.0 |
| *R. leguminosarum* USDA 2370^T^ | 34.5 |
| *R. pisi* DSM 30132^T^ | 38.6 |
| *R. fabae* CCBAU 33202^T^ | 32.2 |
| *R. phaseoli* DSM 30137^T^ | 28.7 |

Table S2. Average Nucleotide Identity using MUMmer (ANIm) between representative strains. (a) Strains from the field population, plus the reference strain 3841. (b) The published sequence of WSM1325, and the type strain of *R. leguminosarum*, USDA2370, compared to TRX34 (genospecies A). Each pairwise comparison was carried out in both directions: rows represent queries compared to the reference genomes that are indicated in the columns.

(a)

(b)

Table S3. Average Nucleotide Identity (ANIm) between published genome sequences of *R. leguminosarum* strains and strains representing each of the five genospecies. Values of 96.0 or above are shaded green; values of 95.0 or above but less than 96.0 are shaded yellow. This table can be opened as an Excel spreadsheet.

Table S4. List of genes present in genospecies B only. All are on either on pRL9 or pRL12, as indicated by the locus tags.

| **Locus tag** | **Position** | **Strand** | **Function** |
| --- | --- | --- | --- |
| pRL90119 | 119494..120420 | - | LysR family transcriptional regulator |
| pRL90120 | 120552..121043 | + | putative 4-carboxymuconolactone decarboxylase |
| pRL90121 | 121201..122367 | - | hypothetical protein |
| pRL90122 | 122532..123563 | - | putative LacI/HTH-type transcriptional regulator |
| pRL90123 | 123678..124775 | + | putative lactose transport ATP-binding protein |
| pRL90124 | 124772..125662 | + | putative transmembrane binding-protein-dependent transporter |
| pRL90125 | 125659..126507 | + | putative permease transporter component |
| pRL90126 | 126570..127898 | + | putative solute binding-protein component of transporter |
| pRL90127 | 128004..130772 | + | putative glycosyl hydrolase |
| pRL90035 | 35946..36167 | - | putative ATP_binding protein of ABC transporter,pseudogene |
| pRL90036 | 36196..36600 | - | putative attachment-related protein |
| pRL90039 | 38011..39597 | + | hypothetical protein |
| pRL90041 | 40760..42379 | - | chaperonin GroEL |
| pRL90043 | 44101..45126 | + | putative transmembrane transport protein |
| pRL90044 | 45123..47843 | + | putative transmembrane ABC transporter |
| pRL90045 | 47845..48966 | + | putative ABC transporter permease component |
| pRL90088 | 89425..89862 | + | hypothetical protein |
| pRL90089 | 89940..90962 | + | hypothetical protein |
| pRL90090 | 90959..91759 | + | putative ATP-binding component of ABC transporter |
| pRL90091 | 91756..92526 | + | putative permease component of ABC transporter |
| pRL90092 | 92660..93400 | - | hypothetical protein |
| pRL90093 | 93687..94118 | + | hypothetical protein |
| pRL90094 | 94115..94597 | + | hypothetical protein |
| pRL90095 | 94783..95190 | - | putative plasmid stability protein |
| pRL90255 | 276794..278197 | - | putative glycine cleavage protein/aminomethyltransferase |
| pRL90256 | 278250..279125 | - | putative 5,10-methylenetetrahydrofolate reductase |
| pRL90257 | 279414..280178 | + | GntR family transcriptional regulator |
| pRL90258 | 280293..281471 | + | putative substrate-binding ABC transporter protein |
| pRL90259 | 281535..282395 | + | putative permease component of ABC transporter |
| pRL90260 | 282392..283438 | + | putative permease component of ABC transporter |
| pRL90261 | 283435..284184 | + | putative ATP-binding ABC transporter |
| pRL90262 | 284181..284903 | + | putative ATP-binding component of ABC transporter |
| pRL120118 | 116168..117097 | + | putative aldo-keto reductase/oxidoreductase |
| pRL120119 | 117411..118196 | - | putative short-chain dehydrogenase |
| pRL120120 | 118244..119017 | - | putative short-chain dehydrogenase |
| pRL120121 | 119028..120362 | - | putative dihydroorotase |
| pRL120122 | 120380..122071 | - | hypothetical protein |
| pRL120123 | 122064..122975 | - | putative polysaccharide deacetylase |
| pRL120124 | 122972..123973 | - | putative NAD-dependent epimerase/dehydratase |
| pRL120125 | 124043..124804 | - | putative short-chain dehydrogenase |
| pRL120126 | 124801..126234 | - | putative D-hydantoinase |
| pRL120127 | 126231..127493 | - | MFS family transporter |
| pRL120128 | 127493..129547 | - | putative ATP-binding component of ABC transporter |
| pRL120129 | 129580..130455 | - | putative permease component of ABC transporter |
| pRL120130 | 130459..131472 | - | putative permease component of ABC transporter |
| pRL120131 | 131542..133221 | - | putative substrate binding component of ABC transporter |
| pRL120132 | 133407..134435 | - | AraC family transcriptional regulator |
| pRL120133 | 134847..135260 | - | putative plasmid stability protein |
| pRL120134 | 135257..135520 | - | putative plasmid stability protein |
| pRL120135 | 135737..136699 | - | putative cyclase |
| pRL120136 | 136751..138124 | - | hypothetical protein |
| pRL120137 | 138144..139352 | - | hypothetical protein |
| pRL120138 | 139349..140389 | - | cobW family cobalamin synthesis protein |
| pRL120139 | 140382..140714 | - | hypothetical protein |
| pRL120140 | 140727..141608 | - | putative imidase |
| pRL120141 | 141662..142780 | - | putative ATP-binding component of ABC transporter |
| pRL120142 | 142879..144114 | - | putative substrate-binding component of ABC transporter |
| pRL120143 | 144218..144991 | - | putative short-chain dehydrogenase/reductase |
| pRL120144 | 145005..145745 | - | putative 3-oxoacyl-[acyl-carrier-protein] reductase (3-ketoacyl-acyl carrier protein reductase) |
| pRL120145 | 145742..146587 | - | putative permease component of ABC transporter |
| pRL120146 | 146589..147488 | - | putative permease component of ABC transporter |
| pRL120147 | 147527..148615 | - | putative ATP-binding component of ABC transporter |
| pRL120148 | 148803..149093 | + | hypothetical protein |
| pRL120149 | 149163..150182 | + | GntR family transcriptional regulator |
| pRL120150 | 150205..151176 | - | putative urea amidolyase related protein |
| pRL120151 | 151166..152041 | - | putative urea amidohydrolyase homologue |
| pRL120152 | 152041..153426 | - | acetyl-CoA carboxylase biotin carboxylase subunit |
| pRL120154 | 153684..154460 | - | hypothetical protein |
| pRL120155 | 154712..155620 | + | LysR family transcriptional regulator |
| pRL120157 | 155971..156909 | - | hypothetical protein |
| pRL120158 | 156921..157889 | - | hypothetical protein |
| pRL120159 | 157902..159188 | - | allantoate amidohydrolase |
| pRL120160 | 159181..159945 | - | DeoR family transcriptional regulator |
| pRL120161 | 160060..161883 | - | putative substrate-binding component of ABC transporter |
| pRL120162 | 161880..162806 | - | putative permease component of ABC transporter |
| pRL120163 | 162803..163783 | - | putative permease component of ABC transporter |
| pRL120164 | 163914..165497 | - | putative component of ABC transporter |

Table S5. Correlation between the utilisation of gamma-hydroxybutyrate and the presence of homologs of genes on plasmid pRL10. Genes with a significant (P < 0.05) positive or negative correlation are listed. PCC = Pearson correlation coefficient.

| Gene | Protein ID | Name | PCC | Product |
| --- | --- | --- | --- | --- |
| Positive correlation | | | | |
| pRL100087 | YP_770380.1 | *acdS* | 0.566 | 1-aminocyclopropane-1-carboxylate deaminase |
| pRL100093 | YP_770383.1 |  | 0.414 | hypothetical protein |
| pRL100103 | YP_770388.1 |  | 0.479 | alcohol dehydrogenase |
| pRL100104 | YP_770389.1 |  | 0.589 | hypothetical protein |
| pRL100105 | YP_770390.1 |  | 0.695 | polyhydroxyalkanoate synthase subunit C |
| pRL100106 | YP_770391.1 |  | 0.495 | hypothetical protein |
| pRL100107 | YP_770392.1 |  | 0.563 | hypothetical protein |
| pRL100109 | YP_770394.1 | *repA* | 0.353 | replication protein RepA |
| pRL100111 | YP_770396.1 |  | 0.289 | hypothetical protein |
| pRL100112 | YP_770397.1 |  | 0.256 | dehalogenase-hydrolase |
| pRL100119 | YP_770400.1 |  | 0.642 | propionate CoA-transferase |
| pRL100120 | YP_770401.1 |  | 0.695 | hypothetical protein |
| pRL100121 | YP_770402.1 | *acsA* | 0.498 | acetyl-coenzyme A synthetase |
| pRL100122 | YP_770403.1 | *adk* | 0.256 | adenylate kinase |
| pRL100124 | YP_770405.1 |  | 0.247 | transposase family protein |
| pRL100125 | YP_770406.1 |  | 0.274 | transposase family protein |
| pRL100128 | YP_770410.1 |  | 0.449 | ATP-binding ABC transporter |
| pRL100129 | YP_770411.1 |  | 0.473 | ABC transporter permease |
| pRL100130 | YP_770412.1 |  | 0.449 | ABC transporter permease |
| pRL100131 | YP_770413.1 |  | 0.449 | ABC transporter substrate-binding protein |
| pRL100132 | YP_770414.1 |  | 0.400 | hypothetical protein |
| pRL100133 | YP_770415.1 | *attJ* | 0.786 | IclR family transcriptional regulatory protein |
| pRL100134 | YP_770416.1 | *attK* | 0.485 | succinate-semialdehyde dehydrogenase |
| pRL100135 | YP_770417.1 | *attL* | 0.811 | 1,3-propanediol dehydrogenase |
| pRL100136 | YP_770418.1 | *attM* | 0.786 | beta lactamase/homoserine lactonase |
| pRL100137 | YP_770419.1 | *metX* | 0.811 | homoserine O-acetyltransferase |
| pRL100138 | YP_770420.1 |  | 0.786 | MerR family transcriptional regulator |
| pRL100146 | YP_770425.1 |  | 0.289 | hypothetical protein |
| pRL100147 | YP_770426.1 |  | 0.256 | hypothetical protein |
| pRL100201 | YP_770479.1 |  | 0.555 | hypothetical protein |
| pRL100202 | YP_770480.1 |  | 0.498 | hypothetical protein |
| Negative correlation | | | | |
| pRL100014 | YP_770316.1 |  | -0.251 | phage integrase |
| pRL100151 | YP_770430.1 | *exsI* | -0.526 | transccriptional regulator |
| pRL100158 | YP_770436.1 | *nifN* | -0.475 | nitrogenase molybdenum-cofactor biosynthesis protein NifN |
| pRL100174 | YP_770453.1 |  | -0.470 | hypothetical protein |
| pRL100175 | YP_770454.1 | *nodO* | -0.475 | nodulation protein |
| pRL100179 | YP_770457.1 | *nodN* | -0.445 | nodulation protein |
| pRL100180 | YP_770458.1 | *nodM* | -0.416 | glucosamine--fructose-6-phosphate aminotransferase |
| pRL100181 | YP_770459.1 | *nodL* | -0.419 | nodulation protein |
| pRL100182 | YP_770460.1 | *nodE* | -0.372 | nodulation protein E |
| pRL100183 | YP_770461.1 | *nodF* | -0.429 | nodulation protein F |
| pRL100184 | YP_770462.1 | *nodD* | -0.471 | nodulation protein D |
| pRL100185 | YP_770463.1 | *nodA* | -0.472 | acyltransferase NodA |
| pRL100187 | YP_770465.1 | *nodC* | -0.283 | N-acetylglucosaminyltransferase |
| pRL100189 | YP_770467.1 | *nodJ* | -0.475 | nodulation protein |
| pRL100194 | YP_770472.1 |  | -0.501 | hypothetical protein |
| pRL100195 | YP_770473.1 | *nifB* | -0.475 | FeMo cofactor biosynthesis protein |
| pRL100196 | YP_770474.1 | *nifA* | -0.387 | nifA transcriptional regulator |
| pRL100197 | YP_770475.1 | *fixX* | -0.389 | ferredoxin-like protein |
| pRL100199 | YP_770477.1 | *fixB* | -0.445 | FixB electron transfer protein |
| pRL100200 | YP_770478.1 | *fixA* | -0.358 | FixA electron transfer protein |
| pRL100210 | YP_770488.1 | *fixI* | -0.256 | transmembrane nitrogen fixation cation transport protein FixI |
| pRL100367 | YP_770643.1 |  | -0.339 | hypothetical protein |

Table S6. Substrate utilisation determined by Biolog. Green (1) = utilised; white (0) = not utilised. The 95 substrates are sorted (left to right) by the number of strains that can use them, the strains are sorted (top to bottom) by genospecies and by major phylogenetic clusters within genospecies. This table can be opened as an Excel spreadsheet.

 Fig. S1. NeighborNet networks based on the sequences that map to each of the 7 replicons in Rlv3841. Colours differentiate the 5 genospecies identified in Fig. 1.

Fig. S2. Correlation between Biolog substrate utilization and the presence of genes in each isolate. The 24 substrates that are utilized by the reference strain 3841 but have variable utilization in the Wentworth population are ranked by the fraction of isolates that utilize them (rows). Genes (columns) were clustered by correlation pattern using an average linkage method, as indicated in the dendrogram at the top.

Fig. S3. NeighborNet phylogeny of the isolates and the reference strain 3841 based on 305 conserved genes. Red symbols indicate biovar *viciae*, blue symbols *trifolii*. Based on the same data and method as Figure 1, but showing strain identifiers.
